# Why should psychiatry be included as examination subject in undergraduate curriculum?

**DOI:** 10.4103/0019-5545.37313

**Published:** 2007

**Authors:** A. B. Ghosh, Asim Kumar Mallick

**Affiliations:** Ex. Associate Professor of Psychiatry, N.R.S. Medical College & Hospital, Kolkata, India; * Associate Professor of Psychiatry, IPGMER, Kolkata, India

The burden of psychiatric morbidity is ever increasing, cutting across regional, socio-economic and cultural barriers.

The global burden of disease due to neuropsychiatric disorders was measured to be 6.8% world wide in 1990. This is projected to increase to 15% by the year 2020. Psychiatric disorders account for 5 of the 10 leading causes of disability across the world.[1]

## PRESENTING SOME ASPECTS OF MENTAL HEALTH IN INDIA

Population - 1081.2 million

Population per sq km - 328.9

Population under 15-32.1%

Population over 60-7.1%

Urban population -28.7%

Men per 100 women - 105

Mental-health disorders account for nearly a sixth of all related disorders. Yet we have just 0.4 psychiatrists and 0.02 psychologists per 100,000 people and 0.25 mental health beds per 10,000 population.

Depression, child abuse, domestic violence, adjustment disorders are on the rise; so is the rate of suicide. The suicide rate in India in the early eighties used to be 6 per 1,00,000 per year. More than a decade later, the rate is 11 per 1,00,000 per year. The highest rate of suicide is prevalent among the younger age groups - between 18 and 29 years.[2]

Despite such high rates of mortality and morbidity associated with psychiatric disorders, the psychiatrist to population ratio in India is very low.

Besides, not all patients with psychiatric disorders or symptoms consult the psychiatrist. The findings of the ICMR-collaborated effort in Bangalore. Hyderabad and Vellore showed that an overwhelming majority of patients with neurosis, depression, alcohol-related problems, sexual problems and psychosomatic disorders seek advice from general physicians and not from the psychiatrists (ICMR, 1982).

Studies have also revealed that nearly two-thirds of all persons who commit suicide contact their family physicians in the month preceding the event. Physicians being aware about psychiatric symptomatalogy therefore, can prevent many untimely deaths.

Patients from all disciplines of Medicine or Surgery may develop psychiatric complications at any point of time.

Delirum, one of the commonest psychiatric diagnoses, is present in nearly 10% of emergency department patients and 40% of all terminally ill patients. Nearly 53% of all patients of chronic renal failure have been shown to have definite psychiatric disturbances. Deafness lasting more than six years is associated with a higher incidence of paranoid schizophrenia.

Depression is an independent risk factor for coronary heart diseases and an indicator of poor prognosis in post myocardial infraction patients.

Psychooncology is one of the expanding facets of oncology research in India and abroad.

Apart from postpartum psychosis, which is a psychiatric emergency, maternal anxiety and depression are definitely known to influence developmental quotient (DQ) of infants, their weight gain and over all morbidity.

Therefore there is an urgent need to sensitize and teach psychiatry in detail to all undergraduate medical students so that they do not falter with or miss psychiatric symptoms in their patients and establish an appropriate liaison with the specialist at the right time.

Unless this is done, important advances made in the different fields of Medicine are likely to go in vain.

In this era of increasing complications of treatment regimens and public awareness, doctor-patient relationships are often at stake. Driven by fear of litigations, doctors too often practice “defensive medicine”, an approach that adds unnecessarily to the public exchequer. Some basic knowledge of into the patients psychology and own reactions may help reduce unnecessary litigations.

The need to train medical students and nonpsychiatrists has been emphasized by various policy making bodies, starting from the Mudaliar Committee (1962) to the National Health Policy (NHP) 1983.

This object can be fulfilled by:

- Thorough training of undergraduate students in Psychiatry and
- Training nonpsychiatrists in the basic principles of psychiatric diagnosis and treatment.

The training of a specialists from other streams can pose problems of time, attitude and other constraints. However, a general practitioners (GPs) training carried out by the ICMR revealed the following difficulties.

1. GPs tend to zealously protect their unique professional freedom and dread to function as an extension of specialists
2. GPs expect their patients to be referred back when they refer them for specialist consultation.

Besides, training of nonpsychiatrists across the country would pose operational difficulties, like bringing all non psychiatrists in government, private, rural, urban sectors under one umbrella. A single short-term training session would hardly be comparable to a rigorous subject training in the M.B.B.S course.

All these factors emphasize the need to train medical students, first and foremost, in the basic principles of Psychiatry. This should include an adequate period of compulsory clinical exposure.

Present status of psychiatry training in the M.B.B.S. course.

- Twelve hours lecture class
- Two weeks clinics
- Theory paper: 1-2 short notes (optional)

### Recommendation:

These evidences urge the need to include psychiatry as an examination subject in under graduate curriculum.

In fact, psychiatry is being taught with due importance globally. Even in our neighbouring countries, psychiatry is an examination subject in the undergraduate curriculum.

If we scrutinize the course and curriculum of Nursing training, we find that they also have given immense importance of psychiatry.
